# The effectiveness of conservative interventions for the management of syndromic hypermobility: a systematic literature review

**DOI:** 10.1007/s10067-020-05284-0

**Published:** 2020-07-17

**Authors:** Shea Palmer, Indi Davey, Laura Oliver, Amara Preece, Laura Sowerby, Sophie House

**Affiliations:** Department of Allied Health Professions, Faculty of Health & Applied Sciences, University of the West of England, Blackberry Hill, Bristol, BS16 1DD UK

**Keywords:** Conservative treatment, Ehlers-Danlos Syndrome, Hypermobility, Systematic review

## Abstract

**Introduction:**

‘Syndromic hypermobility’ encompasses heritable connective tissue disorders such as hypermobile Ehlers-Danlos syndrome and hypermobility spectrum disorders which are characterised by excessive joint range of motion and pain. Conservative interventions such as exercise are the cornerstone of management, yet their effectiveness is unclear.

**Aim:**

To systematically appraise the effectiveness of conservative management for people with syndromic hypermobility.

**Method:**

A systematic online database search was conducted (AMED, BND, CINAHL Plus, MEDLINE, PEDro, PsychINFO and SportDiscus). Potential articles were assessed for eligibility by two researchers against the following criteria: adults and children with a hEDS/HSD diagnosis (or equivalent diagnosis using specific criteria); non-pharmacological or non-surgical interventions; outcomes related to pain, physical function, psychological well-being or quality of life. Controlled trials and cohort studies were included. Critical Appraisal Skills Programme checklists were used to assess methodological quality.

**Results:**

Eleven studies were included, comprising eight controlled trials and three cohort studies. All studies investigated interventions that had exercise as the primary component. Three small controlled studies demonstrated superior effects of conservative management relative to a control group. However, those studies only focused on a single area of the body, only recruited women, and had no long-term follow-up. All studies reported improvements in a wide range of outcomes over time.

**Conclusion:**

Controlled trial evidence for the superiority of conservative management over comparators is weak. There is some evidence that people improve over time. Robust randomised controlled trial research of the long-term effectiveness of ‘whole-body’ (rather than individual joints or body areas) conservative management is required.

**Table:** 

Key Points *• Conservative management is the cornerstone of management of syndromic hypermobility.* *• The review found that evidence for the effectiveness of conservative management relative to no treatment or other conservative comparators was weak.* *• However, there was consistent evidence for effectiveness from pre- to post-treatment.* *• Further robust randomised controlled trial evidence is required.*

## Introduction

‘Hypermobility’ defines the ability of one joint or multiple joints to move beyond what might be considered a normal range of motion [1]. In some cases, hypermobility can be an asset, such as in sport and the performance arts. However, it is also a feature of heritable connective tissue disorders such as hypermobile Ehlers-Danlos syndrome (hEDS) [2] and hypermobility spectrum disorders (HSD) [1]. The terms hEDS/HSD have replaced previous diagnostic categories of Ehlers-Danlos syndrome hypermobility type (EDS-HT) (Villefranche criteria [3]) and joint hypermobility syndrome (JHS) (Brighton criteria [4]). Within this manuscript, we will refer to ‘syndromic hypermobility’ as an umbrella term to cover the new and historical diagnoses. Syndromic hypermobility is commonly associated with pain, fatigue, cycles of injury and recovery [5] in addition to a wide range of other symptoms affecting the musculoskeletal, gastrointestinal, cardiovascular and autonomic nervous systems [6]. Symptoms can have a negative impact upon strength, proprioception, function and quality of life [7]. The prevalence of the new diagnostic categories of hEDS [2] and HSD [1] have yet to be established; however, 30% of people referred to a musculoskeletal triage service in the UK met the historical Brighton criteria for JHS [8]. Similar high prevalence rates for JHS were reported for UK pain management (39.1%), rheumatology (37.0%) and orthopaedic lower limb (10.9%) clinic referrals [9]. Syndromic hypermobility is therefore likely to be more common within musculoskeletal services than traditionally believed, although it should be noted that a much smaller proportion of people are likely to meet the new stricter hEDS diagnostic criteria [2].

Syndromic hypermobility is under-recognised and poorly understood and its assessment and management are deemed complex [1]. A multidisciplinary and patient-centred approach is recommended [10]. The British Society of Paediatric and Adolescent Rheumatology [11] highlighted the ineffectiveness of current medical management for hypermobility-related pain in young children and adolescents, recommending a multi-systemic approach. Surgery should be considered as a last resort when other interventions have been unsuccessful because syndromic hypermobility patients have a higher risk of surgical complications, such as reduced effectiveness of local anaesthesia and delayed wound healing [12]. Rombaut et al. [13] reported that only 33.9% of EDS-HT patients who had surgery reported a positive effect, compared to 63.4% of those receiving physiotherapy. Care needs to be taken in interpreting those figures as the circumstances and indications for such interventions were likely to have been very different and thus they are not directly comparable. Nonetheless, it is clear that the effectiveness of conservative management in people with syndromic hypermobility warrants systematic exploration.

Previous systematic reviews in this area provided some support for the use of conservative management. For example, Smith et al. [14] explored the effectiveness of physiotherapy and occupational therapy management, Palmer et al. [15] investigated the effects of therapeutic exercise, and Peterson et al. [16] reviewed mechanical and physical interventions for lower limb symptoms specifically in children. Findings were mostly positive, with results particularly suggesting that exercise, as a component of management, can be effective in improving symptoms. However, the need for further high-quality evidence was consistently identified, with a lack of randomised controlled trial (RCT) evidence and methodological limitations of the included studies. Several more recent potentially relevant RCTs (e.g. 17, 18) and cohort studies (e.g. 19) have since been published. An updated review that explores a wider range of conservative interventions in both children and adults is therefore required.

Rombaut et al. [13] emphasised the need for evidence-based recommendations for optimal management. For healthcare professionals managing people with EDS, a lack of high-quality clinical guidelines have been reported, limiting evidence-based practice [17]. Palmer et al. [7] highlighted a lack of knowledge and understanding regarding diagnosis and management among physiotherapists. The current systematic review therefore aims to systematically identify and appraise the existing research evidence relating to the effectiveness of conservative management for syndromic hypermobility.

## Methodology

The review was conducted and reported according to PRISMA guidelines [18]. The protocol was not registered with the international prospective register of systematic reviews (PROSPERO).

A librarian with systematic reviewing expertise advised on the choice of electronic databases and construction of the search strategy. The electronic databases chosen were AMED (Allied & Complementary Medicine), CINAHL Plus (Cumulative Index to Nursing & Allied Health Literature), MEDLINE, PsychINFO and SportDiscus (all via EBSCO); BND (British Nursing Database); and PEDro (Physiotherapy Evidence Database).

The research question was refined using the PICO format (Table 1) to ensure a focused and comprehensive search [19]. Consultation with the librarian led to the development of a simple and inclusive search strategy, focussing only on the ‘population’ and ‘intervention’ due to the relatively limited range of literature available in this area (Table 2).

**Table 1:** PICO components associated with the research question

PICO component	Details
Participant (P)	Hypermobile Ehlers-Danlos syndrome (hEDS) or hypermobility spectrum disorders (HSD) (or previous diagnoses of EDS-HT or JHS)
Intervention (I)	Conservative management
Comparison (C)	No intervention, ‘usual care’ or another intervention (randomised controlled trials); or before-after treatment comparison (cohort studies)
Outcome (O)	Pain, physical function, psychological well-being and quality of life

**Table 2:** Final search terms

Search number	Search terms
Search 1	hypermobil* OR Ehlers-Danlos type III OR Ehlers-Danlos type 3
Search 2	treatment OR management OR intervention OR therapy

A Boolean search strategy was used on EBSCO and BND to search for relevant articles. The ‘OR’ operation was used within each search to identify one or more terms, with ‘AND’ being used to combine the search terms [20]. Where possible within each electronic database, the search was limited to articles in the English language published from 1998 onwards. A simplified search strategy of ‘hypermobil*’ was used for PEDro to ensure the retrieval of all relevant articles. The electronic literature search was conducted on 27 February 2019 and updated on 1 June 2020.

Inclusion and exclusion criteria (Table 3) were developed a priori to reflect the PICO research question. Duplicates were removed and the remaining articles were independently evaluated by two researchers against the eligibility criteria to determine the appropriateness of titles, abstracts and full-text articles. Any disagreements between reviewers were discussed by the wider research group and agreed by consensus. All articles included for full-text review and relevant previously published systematic reviews [14–16] were also ‘snowballed’ by one researcher. Snowballing is the process of identifying additional research from reference lists to reduce the risk of missing preliminary evidence [23]. The articles included and excluded, with reasons, were reported according to PRISMA guidelines [18].

**Table 3:** Inclusion and exclusion criteria

Inclusion	Exclusion
hEDS, HSD, EDS-HT, JHS.	Other subtypes of EDS [2, 3].
In adults: specific diagnostic criteria for hEDS [2], HSD [1], EDS-HT [3] or JHS [4]. In children: Beighton score in combination with other symptoms is acceptable as Villefranche [3] and Brighton [4] criteria not validated in children.	Beighton score in isolation.
Adults and children.	Pregnant women as peripheral and pelvic joint laxity increases during pregnancy [21], increasing the prevalence of hypermobility [22].
Conservative management.	Pharmacological or surgical interventions.
Quantitative assessment of pain, physical function, psychological wellbeing or quality of life.	
RCTs and cohort studies, including feasibility, pilot and preliminary studies.	
Peer-reviewed academic journal articles.	
Articles published from 1998 onwards as the international Villefranche (EDS) and Brighton (JHS) criteria were published from 1998.	
English language.	

The methodological quality and risk of bias of the included studies was appraised using Critical Appraisal Skills Programme (CASP) checklists for RCTs [24] and cohort studies [25]. CASP checklists are recommended for group work within healthcare, ensuring succinct and effective consideration of the components needed for critical appraisal of evidence [26]. Two researchers appraised the included articles independently and then agreed a shared interpretation of the quality of each article. Any discrepancies were discussed with the wider research group until consensus was found. The results were then tabulated. Following critical appraisal, key data was extracted (including participants, sample size, location/setting, intervention, outcome measures and findings) and this was also tabulated. This informed a narrative synthesis of the findings. Meta-analysis was not attempted due to substantial heterogeneity in study design, interventions and outcome measures.

## Results

**Fig. 1:**
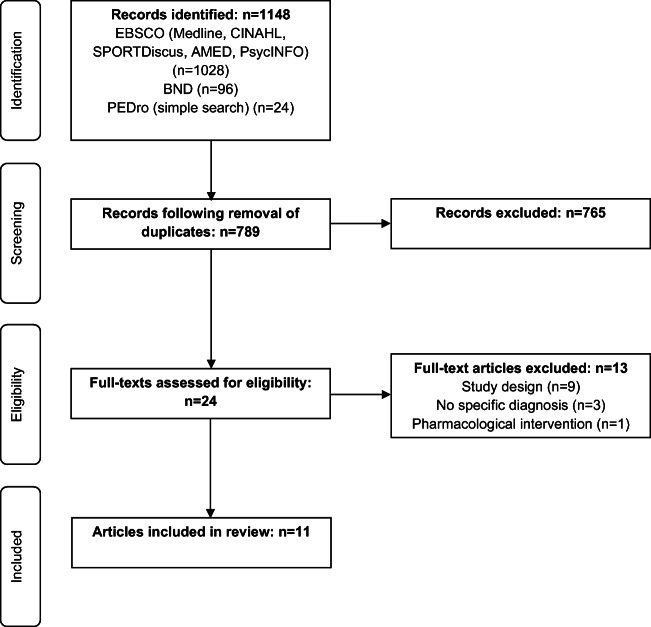
PRISMA flow diagram

Eleven studies were included in the final review. Figure 1 outlines the process of article identification and assessment of eligibility according to PRISMA guidance [18].

Key characteristics of each of the controlled trials and cohort studies are reported in Tables 4 and 5. There were eight controlled trials (including seven RCTs and a pilot RCT) and three cohort studies (one of which was a pilot study).

**Table 4:** Data extraction table describing the key characteristics of each study

Authors Country; setting	Study design	Participants (% female)	Age, years (mean ± SD unless otherwise stated)	Sample size (*n* recruited, *n* completed, sample size calculation)	Diagnostic criteria
Bale et al. (2019) [27]. UK; paediatric rheumatology clinic.	Randomised controlled trial.	Children aged 5–16 years (55% girls).	Intervention: 9.47 ± 3.18, standard care: 9.35 ± 3.20.	*n* = 119 recruited (intervention: *n* = 59, standard care: *n* = 60). *n* = 105 completed (intervention *n* = 54, standard care *n* = 51) (12% attrition). Prospective sample size calculation *n* = 100 (*n* = 50 per arm).	Beighton score ≥ 4 or Bulbena score ≥ 5 (males) or ≥ 6 (females). Plus musculoskeletal pain in ≥ 1 body area for ≥ 3 months.
Bathen et al. (2013) [28]. Norway; rehabilitation hospital.	Pilot cohort study.	Adults (100% women).	Median, range = 35, 20–51.	*n* = 12 recruited. *n* = 12 completed (0% attrition). No prospective sample size calculation (pilot study).	Villefranche criteria (EDS-HT) or Brighton criteria (JHS).
Celenay & Kaya (2017) [29]. Turkey; university physiotherapy and rehabilitation department.	Randomised controlled trial.	Adults aged 18–30 years (100% women).	Exercise: 20.3 ± 2.2, Control: 21.0 ± 2.2.	*n* = 46 recruited (exercise *n* = 23, control *n* = 23). *n* = 38 completed (exercise *n* = 20, control *n* = 18) (17% attrition). Prospective sample size calculation *n* = 42 (*n* = 21 per arm).	Brighton criteria (JHS).
Daman et al. (2019) [30]. Iran; physiotherapy, orthopaedic knee and rheumatology clinics.	Randomised controlled trial.	Adults aged 18–30 years (100% women).	Exercise: 22.25 ± 1.28, Control: 21.66 ± 1.96.	*n* = 24 recruited (exercise *n* = 12, control *n* = 12). *n* completed not explicitly reported (% attrition unable to assess). Prospective sample size calculation *n* = 24.	Brighton criteria (JHS).
Ferrell et al. (2004) [31]. UK; hypermobility clinic.	Cohort study.	Adults (89% women).	Mean, range = 27.3, 16–49.	*n* = 20 recruited. *n* = 18 completed (10% attrition). No prospective sample size calculation.	Brighton criteria (JHS) plus knee pain.
Kemp et al. (2010) [32]. UK; children’s rheumatology department.	Randomised controlled trial.	Children aged 7–16 years (33% girls).	Targeted physiotherapy: 11.0 ± 2.5, generalised physiotherapy: 10.7 ± 2.5.	*n* = 57 recruited (targeted *n* = 30, generalised *n* = 27). *n* = 32 completed (targeted *n* = 17, generalised *n* = 15) (44% attrition). Prospective sample size calculation *n* = 96 (*n* = 48 per arm).	Brighton criteria (JHS) plus arthralgia for > 3 months.
Pacey et al. (2013) [33]. Australia; children’s hospital clinics.	Randomised controlled trial.	Children aged 7–16 years (66% girls).	Training in hypermobile range (‘hypermobile’): 13.48 ± 3.05, training to neutral (‘neutral’) 11.02 ± 2.51 (*p* = 0.04).	*n* = 29 recruited. *n* = 26 randomised (hypermobile *n* = 12, neutral *n* = 14) *n* = 25 completed (hypermobile *n* = 11, neutral *n* = 14) (4% attrition). Prospective sample size calculation *n* = 26 (*n* = 13 per arm).	Knee pain, Beighton score ≥ 5, > 10° knee hyperextension. Brighton criteria (JHS).
Palmer et al. (2016) [34]. UK; two rheumatology physiotherapy services.	Pilot randomised controlled trial.	Adults aged ≥ 16 years (90% women).	Advice and physiotherapy: 37.2 ± 14.13, advice: 33.3 ± 9.71 years.	*n* = 29 recruited (advice and physiotherapy *n* = 15, advice *n* = 14). *n* = 19 completed (advice and physiotherapy *n* = 11, advice *n* = 8) (34% attrition). No prospective sample size calculation (pilot study).	Brighton criteria (JHS).
Reychler et al. (2019) [35]. Belgium; rheumatology outpatient ward.	Randomised controlled trial.	Adults (100% women).	Intervention: 45.8 ± 5.5, control: 53.1 ± 3.4 (*p* = 0.011).	*n* = 20 recruited (intervention *n* = 10, control *n* = 10). *n* = 19 completed (intervention *n* = 9, control *n* = 10) (5% attrition). Prospective sample size calculation *n* = 20 (*n* = 10 per arm).	Villefranche criteria (EDS-HT) and Brighton criteria (JHS). Reviewed against 2017 criteria to confirm hEDS diagnosis.
Sahin et al. (2008) [36]. Turkey; physical medicine and rehabilitation department outpatient clinic.	Randomised controlled trial.	Adults aged 20–45 years (85% women). No prospective sample size calculation. *n* = 40 recruited	Exercise: 25.60 ± 5.98, control: 27.68 ± 7.77.	*n* = 40 recruited (exercise *n* = 15, control *n* = 25). *n* = 40 completed (exercise *n* = 15, control *n* = 25) (0% attrition). No prospective sample size calculation.	Brighton criteria (JHS) plus knee pain.
To & Alexander (2018) [37]. UK; patient support groups and hospital group; and university, local sports centres, and clubs.	Cohort study.	Adults aged 18–55 years (84% women).	JHS: 34.6 ± 10.6, GJH: 31.8 ± 10.5, Control: 38.2 ± 9.3.	*n* = 102 recruited (JHS *n* = 47, GJH *n* = 29, control *n* = 26). *n* = 72 completed (JHS *n* = 31, GJH *n* = 20, control *n* = 21) (29% attrition). Prospective sample size calculation *n* = 60 (*n* = 20 per arm).	Brighton criteria with anterior knee pain (JHS group). Beighton score ≥ 4 with anterior knee pain (GJH group). Beighton score ≤ 3 with anterior knee pain (control group).

*EDS-HT* Ehlers-Danlos syndrome hypermobility type, *GJH* generalised joint hypermobility, *hEDS* hypermobile Ehlers-Danlos syndrome, *JHS* joint hypermobility syndrome, *SD* standard deviation, *UK* United Kingdom of Great Britain & Northern Ireland

**Table 5:** Data extraction table describing the key characteristics of the interventions, outcome measures and findings from each study

Authors	Study design	Intervention details	Outcome measures	Main statistical findings	Authors’ conclusion
Bale et al. (2019) [27].	Randomised controlled trial.	Duration: 8 weeks. Intervention: individual multidisciplinary programme of 3 individualised PT sessions, OT/PT school visit, OT home assessment, equipment provision. Standard care: single paediatric rheumatology appointment, verbal advice, referral to PT/OT if required.	Follow-up time points: 3 months and 12 months following baseline. Child-reported pain: Wong-Baker faces pain scale. Parent-reported pain: VAS. Parent-reported physical function: CHAQ. Child-reported QoL: Child Health Utility. Motor skills and coordination: Movement Assessment Battery for Children. Grip strength: hand-held dynamometer.	Effect of group: No significant differences between groups at 3 months or 12 months for any outcome (all *p* > 0.05). Effect of time: significant improvements from baseline to 12 months in child-reported pain; parent-reported pain; motor skills and coordination; and grip strength (data from both groups combined) (all *p* < 0.05). No significant changes from baseline to 12 months in parent-reported physical function or child-reported QoL (data from both groups combined) (both *p* > 0.05).	“We conclude that… standard care… is sufficient to lead to a sustained improvement in symptoms over a twelve-month period and that a more intensive multidisciplinary approach offers little further benefit.”
Bathen et al. (2013) [28].	Pilot cohort study.	Duration: 15 weeks. Intervention: underpinned by a cognitive behavioural approach. In-patient programme (2.5-week duration) included testing, physical training, group discussions and lectures. Home exercise (3-month duration) focused on strength and endurance, core stability, body awareness, posture and physical endurance, facilitated weekly by local PT. Readmission (4 days duration) for retesting and further training advice.	Follow-up time point: 15 weeks. Daily activity limitations: COPM (activity performance and performance satisfaction). Muscle strength and endurance: tandem walking backwards, stair walking up and down, and stepping up on toes. Kinesiophobia: Tampa Scale of Kinesiophobia-13. Pain intensity over last 7 days: Numerical Pain Rating Scale.	Effect of group: N/A. Effect of time: significant improvements from baseline to 15 weeks in activity performance; performance satisfaction; tandem walking backwards; stair walking up; up on toes; and kinesiophobia (all *p* < 0.05). No significant improvements from baseline to 15 weeks in stair walking down or pain intensity (both *p* > 0.05).	“…an intensive multidisciplinary rehabilitation program with a cognitive behavioural approach, including intensive muscle strength and –endurance training and pain coping might be feasible and safe for adults with EDS-HT/JHS.”
Celenay & Kaya (2017) [29].	Randomised controlled trial.	Duration: 8 weeks. Intervention: 40–45-min group exercise programme (10 min warm-up, 25 min spinal stabilisation exercises, 5–10-min cool-down and stretching). 3 days per week for 8 weeks. Supervised by an experienced physiotherapist. Maximum 5–6 participants per group. Control: no intervention.	Follow-up time point: 8 weeks. Pain intensity: VAS. Trunk endurance: McGill’s trunk muscle endurance tests (× 4 parameters). Postural stability: Biodex Balance System (× 4 parameters).	Effect of group: Significant difference between groups (in favour of intervention) for pain intensity (*p* = 0.022); all 4 trunk endurance tests (all *p* < 0.05); and 1 of the postural stability tests (dynamic mode eyes open, *p* = 0.036). No significant difference between groups in the other 3 postural stability tests (all *p* > 0.05). Effect of time: Significant improvements in the intervention group from baseline to 8 weeks in pain intensity (*p* = 0.001); all 4 trunk endurance tests (all *p* < 0.05); and 2 of 4 postural stability tests (static mode eyes closed, *p* = 0.028; dynamic mode eyes closed, *p* = 0.008). No significant improvements in the intervention group from baseline to 8 weeks in the other 2 postural stability tests (both *p* > 0.05). No significant improvements in the control group from baseline to 8 weeks for any outcome.	“This exercise program can be used for general pain relief, trunk muscle endurance weakness, and postural impairment in women with BJHS.”
Daman et al. (2019) [30].	Randomised controlled trial.	Duration: 4 weeks. Intervention: combined exercise therapy. Closed kinetic chain and proprioception exercises with progression. 3 days per week for 4 weeks. Supervised by an expert physiotherapist. Control: no intervention.	Follow-up time point: 4 weeks. Pain intensity: VAS. QoL: SF-36 (physical functioning and mental health). Knee joint position sense: angle error using goniometry (weight-bearing and non-weight-bearing).	Effect of group: Significant difference between groups at 4 weeks (in favour of intervention) for joint position sense in weight-bearing (*p* = 0.03) and non-weight-bearing (*p* = 0.009); pain intensity (*p* < 0.001); and physical functioning (*p* = 0.01). No significant difference between groups at 4 weeks for mental health (*p* = 0.42). Effect of time: Significant improvements in the intervention group from baseline to 4 weeks in joint position sense in weight-bearing (*p* = 0.005) and non-weight-bearing (*p* = 0.01); pain intensity (*p* = 0.001); and physical functioning (*p* = 0.01). No significant improvement in the intervention group from baseline to 4 weeks in mental health (*p* > 0.05). No significant improvements from baseline to 4 weeks in the control group for any outcome (*p* > 0.05).	“…combined exercise therapy reduced pain intensity, and improved joint proprioception and quality of life immediately after the intervention.”
Ferrell et al. (2004) [31].	Cohort study.	Duration: 8 weeks. Intervention: home exercise programme with progressive, closed kinetic chain exercises for knee proprioception, balance and strength. 4 days per week for 8 weeks.	Follow-up time point: 8 weeks. Knee joint proprioception: threshold detection paradigm. Balance: instrumented balance board. Muscle strength: Kin-Com isokinetic dynamometer. Pain intensity: VAS. QoL: SF-36 (physical functioning and mental health).	Effect of group: N/A. Effect of time: Significant improvements from baseline to 8 weeks in all outcomes (all *p* < 0.05).	“Appropriate exercises lead not only to symptomatic improvement, but also to demonstrable enhancement of objective parameters such as proprioception.”
Kemp et al. (2010) [32].	Randomised controlled trial.	Duration: 6 weeks. Targeted physiotherapy: standardised exercises of symptomatic joints aimed at addressing functional stability. Generalised physiotherapy: standardised general exercises aimed at maximising muscle strengthening and fitness. Both interventions involved 6 individual 30-min weekly sessions provided by a senior physiotherapist. Both groups also received home exercises to be done on a daily basis.	Follow-up time points: Approximately 2 months and 5 months following randomisation. Pain intensity (child-reported): faces scale < 11 years and VAS ≥ 11 years. Pain intensity (parent-reported): VAS. Global evaluation of impact (parent-reported): VAS. Functional impairment: CHAQ. General physical condition: 6-min shuttle test.	Effect of group: no significant differences between groups at 2 months in any outcome (all *p* > 0.05). Significant difference between groups at 5 months (in favour of targeted physiotherapy) for parental global assessment (*p* = 0.027). No significant differences between groups at 5 months in any other outcome (all *p* > 0.05). Effect of time: significant improvements from baseline to 2 months in child-reported pain (*p* < 0.001); parent-reported pain (*p* < 0.001); parental global assessment (*p* = 0.005); and CHAQ (*p* = 0.024) (data from both groups combined). No improvement from baseline to 2 months in shuttle test (*p* = 0.42) (data from both groups combined). Significant improvements from baseline to 5 months in child-reported pain (*p* < 0.001); parent-reported pain (*p* < 0.001); and CHAQ (*p* = 0.035) (data from both groups combined). No improvement from baseline to 5 months in parental global assessment (*p* = 0.13) (data from both groups combined). Shuttle test not assessed at 5 months.	“…significant and sustained reduction in pain when both groups were combined, but did not detect any difference between the groups.”
Pacey et al. (2013) [33].	Randomised controlled trial.	Duration: 8 weeks. Both groups received the same knee exercises with the only difference being the range in which the exercises were carried out (hypermobile into full knee hyperextension, Neutral to neutral knee extension only). Exercises included quadriceps and hamstring isometric exercises; theraband resisted and joint exercises in standing; eccentric hamstring strengthening; and gluteus medius and hip abductor strengthening. Exercises were progressed by increasing repetitions, resistance and complexity. 6 physiotherapy sessions over 8 weeks (weekly for 4 weeks and fortnightly for 4 weeks). Each session lasted 30–60 min, provided by an experienced paediatric physiotherapist. Both groups also received home exercises to be performed 5 times per week.	Follow-up time point: 8 weeks. Average and maximum knee pain over the last week (child-reported): VAS. Global impression of change (child-reported): PGIC. Functional ability (child-reported): CHAQ. QoL (parent-reported): CHQ (physical and psychosocial summary scores). Thigh muscle strength: hand-held dynamometer. Stair ascent/descent for 2 min: number of flights completed.	Effect of group: No significant differences between groups at 8 weeks in any child-reported or physical outcome measure (all *p* > 0.05). Significant difference between groups at 8 weeks (in favour of Neutral) for parent-reported physical CHQ (*p* = 0.037); and (in favour of Hypermobile) for parent-reported psychosocial CHQ (*p* = 0.009). Effect of time: Significant improvements from baseline to 8 weeks in average and maximum child-reported pain (*p* = 0.004 and 0.003); PGIC (*p* < 0.001); thigh strength (*p* = 0.004); and parent-reported physical and psychosocial CHQ (*p* = 0.002 and 0.03) (data from both groups combined). No improvements from baseline to 8 weeks in CHAQ (*p* = 0.433) or stair ascent/descent (*p* = 0.11) (data from both groups combined).	“… a physiotherapist-supervised exercise programme is significantly effective in reducing pain, improving health-related quality of life, and increasing muscle strength in children with JHS and knee pain.”
Palmer et al. (2016) [34].	Pilot randomised controlled trial.	Duration: 4 months. Advice: tailored information and advice on self-management, including patient organisation booklets. Advice was a one-off session delivered by a trained therapist. Advice and physiotherapy: Advice as above plus comprehensive whole-body physiotherapy intervention. Physiotherapy delivered by a trained therapist via 6 × 30-min sessions across 4 months.	Follow-up time points: 4 months and 7 months following baseline. Impact: BIoH questionnaire. Pain: VAS. Disease activity: MDHAQ (including RAPID3). QoL: EQ-5D-5L. Adverse events. Qualitative interviews with therapists and people with JHS.	Effect of group: no statistical analysis (pilot study). Effect of time: no statistical analysis (pilot study). Mean improvements in both groups from baseline to 4 months in BIoH scores. Continued improvement from 4 months to 7 months in the Advice & Physiotherapy group in BIoH score, whilst the Advice group reverted to baseline. Results support the feasibility of a RCT.	“A comprehensive physiotherapy intervention package was developed which was generally very well received by both patients and physiotherapists, and shows evidence of promise in improving the impact of JHS.”
Reychler et al. (2019) [35].	Randomised controlled trial.	Duration: 6 weeks. Intervention: inspiration exercises using a pressure threshold IMT device. 5 unsupervised sessions per week for 6 weeks. Each session included 6 × 10 repetitions. Progressive increase in resistance from 60% to 85% of initial maximal SNIP. Control: not explicitly reported.	Follow-up time point: 7 weeks. Inspiratory muscle strength: maximal SNIP using pressure meter. Cardiopulmonary function: FVC and FEV1. Functional exercise capacity: 6MWD. [Sp0_2_, dyspnoea (VAS) and heart rate assessed before and after 6MWD]. Anxiety & depression: HADS. Physical activity: Baecke questionnaire.	Effect of group: Significant difference between groups at 7 weeks (in favour of intervention) for SNIP (*p* < 0.001), FEV1 (*p* = 0.009) and 6MWD (*p* = 0.003). No significant differences between groups at 7 weeks in any other outcome measure (all *p* > 0.05). Effect of time: Significant improvements in the intervention group from baseline to 7 weeks in SNIP (*p* = 0.003), FEV1 (*p* = 0.010) and 6MWD (*p* = 0.036). Significant increase in the intervention group from baseline to 7 weeks in pre- to post-6MWD heart rate variation (*p* = 0.029). Significant decrease in the control group from baseline to 7 weeks in FVC (*p* = 0.047). No significant changes in either group from baseline to 7 weeks for any other outcome (all *p* > 0.05).	“The IMT protocol used in this randomized controlled trial achieved clinically significant increases in both respiratory and exercise capacity…”
Sahin et al. (2008) [36].	Randomised controlled trial.	Duration: 8 weeks. Intervention: proprioception exercises (including balance and kinaesthesia exercises). Supervised by a doctor in clinic 3 days per week for 8 weeks. Control: no intervention.	Follow-up time point: 8 weeks. Joint position sense: active knee joint reproduction test using isokinetic dynamometer. Pain intensity on movement and at rest: VAS. Function: AIMS-2	Effect of group: no between-group statistical comparisons reported at 8 weeks. Effect of time: Significant improvements from baseline to 8 weeks in the intervention group in pain on movement (*p* = 0.010); pain at rest (*p* = 0.027); joint position sense (left knee *p* = 0.001, right knee *p* = 0.000); and the occupational activity subscale of AIMS-2 (*p* < 0.05). No significant improvement from baseline to 8 weeks in the intervention group in the other AIMS-2 subscales (all *p* > 0.05). No significant improvement from baseline to 8 weeks in the control group in any outcome (all *p* > 0.05).	“…proprioception exercises cause decrease in pain and improvement of functional status in BJHS group.”
To & Alexander (2018) [37].	Cohort study.	Duration: 16 weeks. Intervention: All participants (JHS, GJH, control) received individualised leg exercises. Exercises were goal-related and functional where possible. American College of Sports Medicine guidelines were followed for progression. Appointment with a senior physiotherapist every 2 weeks for 16 weeks. Home exercises 3 times per week on non-consecutive days.	Follow-up time point: 16 weeks. Intention to treat analysis using multiple imputation to account for missing data. Pain intensity: VAS. Pain medication: diary. Knee function: Lysholm knee scale. Activity: Adjusted Activity Score and Human Activity Profile score. Muscle strength: concentric and eccentric leg press torque using isokinetic dynamometer.	Effect of group: no difference between groups in the rate of change in concentric and eccentric torque from baseline to 16 weeks (*p* > 0.05). Significant differences between groups in concentric and eccentric torque at baseline (JHS < Control < GJH). Estimated to take 11.6 weeks (concentric torque) and 16.2 weeks (eccentric torque) for JHS to reach the baseline GJH mean. Effect of time: Significant improvements in the JHS group from baseline to 16 weeks in pain (*p* < 0.00); knee function (*p* < 0.00); and activity (*p* < 0.00). All improvements in the JHS group were clinically meaningful.	“People with JHS can strengthen at the same rate as other people in pain… Their increase in strength related to their decrease in pain.”

*6MWD* 6-minute walk distance, *AIMS-2* Arthritis Impact Measurement Scales-2, *BIoH* Bristol Impact of Hypermobility questionnaire, *BJHS* benign joint hypermobility syndrome, *CHAQ* Childhood Health Assessment Questionnaire, *CHQ* Child Health Questionnaire, *COPM* Canadian Occupational Performance Measure, *EDS-HT* Ehlers-Danlos syndrome hypermobility type, *EQ-5D-5L* EuroQol-5 Dimensions-5 Levels questionnaire, *FEV1* forced expiratory volume in 1 s, *FVC* forced vital capacity, *gjh* generalised joint hypermobility, *HADS* Hospital Anxiety & Depression Scale, *IMT* inspiratory muscle training, *JHS* joint hypermobility syndrome, *MDHAQ* Multidimensional Health Assessment Questionnaire, *N/A* not applicable, *OT* occupational therapy, *PGIC* patient’s global impression of change, *PT* physiotherapy, *QoL* quality of life, *RAPID3* routine assessment of patient index data, *RCT* randomised controlled trial, *SF-36* Short-Form 36 questionnaire, *SNIP* sniff nasal inspiratory pressure, *Sp0*_*2*_ oxygen saturation, *VAS* Visual Analogue Scale

Of the controlled studies, only Celenay and Kaya [29], Daman et al. [30] and Reychler et al. [35] found consistent evidence of the superiority of conservative interventions relative to no-treatment controls post-treatment. However, those studies only focused on a single area of the body, only recruited women, and had no long-term follow-up. Unfortunately, Sahin et al. [36] failed to report a direct head-to-head statistical comparison between trial arms following treatment. Two controlled studies [32, 33] reported inconsistent findings, with only parent-reported outcomes demonstrating differences between groups. For example, Pacey et al. [33] found that the parent-reported physical quality of life score favoured exercise to neutral, whilst the parent-reported psychosocial score favoured exercise into the hypermobile range. Kemp et al. [32] found that parental global assessment favoured targeted physiotherapy rather than generalised physiotherapy, but only at 5 months. None of the other child-reported or physical outcome measures reported by these studies [32, 33] differed between groups. Bale et al. [27] found no difference on any outcome between a multidisciplinary intervention and usual care. The final controlled trial [34] was clearly identified as a pilot study, aimed at informing a future definitive trial of physiotherapy. It was therefore not explicitly designed to determine the effectiveness of the intervention and, as a result, did not conduct between-group statistical analyses. In summary, the evidence for the effectiveness of conservative management relative to comparators is weak and contradictory.

There was much more consistent evidence from the controlled studies and cohort studies of positive effects of conservative management from pre- to post-treatment. This included a very wide variety of patient-reported, parent-reported and objective outcomes across impairment, activity and participation levels.

Five studies were conducted in the UK, two in Turkey, and one each in Australia, Iran and Norway. Three studies were with children [27, 32, 33], with the remainder being in adult participants. Bathen et al. [28], Celenay and Kaya [29], Daman et al. [30] and Reychler et al. [35] recruited only women, whilst the others had both sexes. Total samples sizes recruited ranged from *n* = 12 to *n* = 119, with *n* = 10 to *n* = 59 allocated to the conservative intervention groups.

The intervention duration ranged from 4 weeks to 4 months, with the final outcomes being at the end of treatment in seven of the 11 studies. Bale et al. [27] had the longest follow-up (12 months following baseline). All conservative interventions featured exercise as a core component, accompanied in some cases by a range of additional interventions such as occupational therapy [27], discussions and lectures [28] or information and advice [34]. Some interventions (and associated outcomes) were specific to a single body area such as the spine [29], inspiratory muscles [35] or the knee joint [18, 19, 30, 32, 35]. A very wide range of impairment, activity and participation level outcome measures were included across the studies.

Summaries of the CASP appraisal of each of the RCTs and cohort studies are reported in Table 6 and Table 7, respectively. Key aspects related to study quality will be discussed in the following section.

**Table 6:** Critical Appraisal Skills Programme checklist for randomised controlled trials. ✓ = Yes, ✗ = No, ? = Can’t Tell, CIs = Confidence Intervals

CASP Checklist Question	Bale et al. (2019) [17]	Celenay & Kaya (2017) [28]	Daman et al. (2019) [18]	Kemp et al. (2010) [31]	Pacey et al. (2013) [32]	Palmer et al. (2016) [33]	Reychler et al. (2019) [29]	Sahin et al. (2008) [30]
1. Did the trial address a clearly focused issue?	✓	✓	✓	✓	✓	✓	✓	✓
2. Was the assignment of patients to treatments randomised?	✓	✓	✓	✓	✓	✓	✓	✓
3. Were all of the patients who entered the trial properly accounted for at its conclusion?	✓	✓	?	✓	✓	✓	✓	✓
4. Were patients, health workers and study personnel ‘blind’ to treatment?	✓	✓	✓	✓	✓	✗	✓	✗
5. Were the groups similar at the start of the trial?	✓	✓	✓	✓	✗	✓	✗	✓
6. Aside from the experimental intervention, were the groups treated equally?	✗	✓	?	✓	✓	✓	?	?
7. How large was the treatment effect?	See Table 5	See Table 5	See Table 5	See Table 5	See Table 5	See Table 5	See Table 5	See Table 5
8. How precise was the estimate of the treatment effect?	95% CIs reported	No CIs reported	No CIs reported	95% CIs reported	95% CIs reported	95% CIs eported	No CIs reported for between group differences	No CIs reported
9. Can the results be applied to the local population, or in your context?	✓	✗	✗	✓	✓	✗	✓	?
10. Were all clinically important outcomes considered?	✓	✓	✓	✗	✗	✓	✓	✗
11. Are the benefits worth the harms and costs?	✓	✓	✓	✗	✓	✓	✓	✓

**Table 7:** Critical Appraisal Skills Programme checklist for cohort studies. ✓= Yes, ✗ = No, ? = Can’t Tell, CIs = Confidence Intervals

CASP Checklist Question	Bathen et al. (2013) [34]	Ferrell et al. (2004) [35]	To & Alexander (2018) [19]
1. Did the trial address a clearly focused issue?	✓	✓	✓
2. Was the cohort recruited in an acceptable way?	?	?	✓
3. Was the exposure accurately measured to minimise bias?	✗	✗	✓
4. Was the outcome accurately measured✗to minimise bias?	✓	✓	✓
5(a) Have the authors identified all important confounding factors?	?	?	✓
5(b) Have they taken account of the confounding factors in the design and/or analysis?	✗	✗	✓
6(a) Was the follow-up of subjects complete enough?	✗	✗	✗
6(b) Was the follow-up of subjects long enough?	✗	✗	✗
7. What are the results of this study?	See Table 5	See Table 5	See Table 5
8. How precise are the results?	No CIs reported	No CIs reported	95% CIs reported
9. Do you believe the results?	✗	✗	✓
10. Can the results be applied to the local population?	✗	✓	✓
11. Do the results of this study fit with other available evidence?	✓	✗	✗
12. What are the implications of this study for practice?	See Discussion section	See Discussion section	See Discussion section

## Discussion

This review aimed to determine the effectiveness of conservative management for the management of syndromic hypermobility. The included papers all used exercise as a core component of the interventions investigated. Post-treatment, there was some weak evidence for superior effects of conservative management relative to no-treatment controls [18, 28, 29], but such evidence was inconsistent or absent when compared against other forms of conservative management [27, 32, 33, 36]. All studies reported evidence of positive effects on a range of outcomes from pre- to post-treatment within the conservative intervention groups. However, the studies included in the review had a range of methodological limitations and thus the evidence needs to be interpreted with caution.

### Study quality

The overall quality of the controlled studies was variable (Table 6), with identified weaknesses in relation to factors such as blinding, the comparability of groups at the start of the trial, the comparability of how groups were treated, the reporting of precision estimates, and the completeness of outcome assessment. The cohort studies also had identified weaknesses, particularly in relation to the identification of and accounting for confounding variables and the completeness and length of follow-up (Table 7). Some of the methodological limitations will now be explored in more detail.

#### Group allocation

Randomisation reduces systematic error and improves internal validity by ensuring that group differences following intervention are due to treatment effects and not confounding variables [38]. Random allocation also aims to reduce selection bias by distributing patient characteristics evenly between groups [39].

Randomisation was conducted in all eight of the controlled trials. Kemp et al. [32] and Celenay and Kaya [29] both used computer-generated block randomisation, whilst Daman et al. [30], Reychler et al. [35], Pacey et al. [33] and Palmer et al. [34] used simple computer-generated randomisation. Bale et al. [27] used minimisation techniques, a valid alternative to randomisation [40]. However, the study by Sahin et al. [36] lacked clarity and detail regarding the randomisation process.

Of the controlled trials, Bale et al. [27], Celenay and Kaya [29], Daman et al. [30], Kemp et al. [32] and Sahin et al. [36] reported that randomisation resulted in no significant differences between group characteristics at baseline. However, Reychler et al. [35] and Pacey et al. [33] found a difference in age and Palmer et al. [34] found a difference in age and sex between groups following randomisation.

Due to the study design, randomisation was not appropriate in the three cohort studies. Ferrell et al. [31] and Bathen et al. [28] conducted single group cohort studies, in which all participants were exposed to the same intervention, whereas To and Alexander [37] conducted a three-group cohort study (JHS, generalised joint hypermobility (GJH) and normal mobility). Cohort studies can be advantageous in collecting specific exposure data; however, they are often criticised for being vulnerable to influences from confounding variables [41].

#### Blinding

Six of the eight controlled trials reported blinding. Pacey et al. [33] performed a double-blind trial, with the treating therapist blinded to the results of assessment and patients blinded to the difference between the two exercise programmes. Bale et al. [27], Daman et al. [30], Celenay and Kaya [29] and Reychler et al. [35] performed single-blind studies, blinding outcome assessors to intervention groups. Kemp et al. [32] also used a single-blind method, blinding the physiotherapist delivering sessions to participant demographic data, diagnostic criteria, symptom scores, joint range, strength and fitness assessments.

Palmer et al. [34] reported being unable to blind participants or assessors due to the nature of the intervention. This is recognised as a limitation in evaluating conservative treatments [42, 43]. Blinding was not discussed or reported by Ferrell et al. [31], Sahin et al. [36], To and Alexander [37] or Bathen et al. [28].

#### Confounding variables

Confounding variables are external factors that affect the true relationship between an intervention (the independent variable) and the study outcome (the dependent variable) [44]. Consideration of confounding variables is particularly important in cohort studies as they reduce the internal validity of the study [45]. To and Alexander [37] recognised that pain was likely to confound their investigation, and therefore ensured that anterior knee pain was a feature of all groups. Detailed eligibility criteria were also documented, which reduces the risk that the recruited population had other confounding variables that may negatively affect the outcomes [46]. However, Bathen et al. [28] and Ferrell et al. [31] both lacked detail regarding the presence or control of confounding variables, meaning that external factors, such as comorbidities, may have influenced findings. Bathen et al. [28] reported only the demographics and characteristics of the participants, and Ferrell et al. [31] reported only their age and Beighton score. If confounding variables are not controlled for during selection, they can be accounted for during statistical analysis [46]. However, neither Bathen et al. [28] nor Ferrell et al. [31] reported doing so, limiting interpretation of their findings as the effect of the interventions may have been obscured by external factors [47].

#### Participants

The majority of studies used convenience sampling to recruit participants from hospital physiotherapy, rheumatology or hypermobility clinics. Convenience sampling refers to selecting participants based on accessibility; although considered the least rigorous sampling method, it is widely used within clinical research as it is easy and affordable [48, 49]. To and Alexander [37] recruited from a variety of sources, including support groups. Such sources risk recruitment of an unrepresentative sample as support group members are likely to be more compliant and proactive in managing their condition [50]. Bathen et al. [28] did not clearly state from where participants were recruited.

Clear eligibility criteria were outlined in nine studies, with the exception of Bathen et al. [28] and Ferrell et al. [31]. It is essential to consider how eligibility criteria might impact the validity of the research [51]. For example, very strict eligibility criteria can limit external validity [52]. Six of the nine studies that clearly reported eligibility criteria excluded participants that had a history of other musculoskeletal pathology, including osteoarthritis, previous surgery and ligament damage, particularly at the knee joint. The remaining three studies excluded participants based on refusal to give consent or the presence of other chronic conditions. Exclusion based on comorbidities is likely to strengthen claims of a causal path between exposure and outcome [53]. However, patients with syndromic hypermobility are at higher risk of musculoskeletal complications, chronic pain and joint degeneration [54]. Therefore, exclusion of comorbidities may create a sample that is unrepresentative, limiting external validity and clinical relevance.

#### Sample size and retention

Researchers use sample size calculations to determine how many participants are required to answer their research question [55]. An adequate sample size is required to detect statistically significant treatment effects [56]. Small sample sizes are vulnerable to type two statistical errors, and larger than required sample sizes risk wasting limited resources [57]. Of the eight controlled trials, only two [27, 30] performed prospective sample size calculations and then managed to recruit and retain the number of participants identified. The other six controlled trials either did not report a prospective sample size [34, 36], failed to recruit the required sample [32, 33] or recruited to the required sample but were unable to retain that number in the trial [29, 35]. Of the three cohort studies, only To and Alexander [37] recruited and retained participants in excess of a prospectively calculated sample size. The others did not report prospective sample size calculations [28, 31].

Although Bathen et al. [28], Sahin et al. [36] and Ferrell et al. [31] did not perform sample size calculations, they all found statistically significant improvements in some outcomes over time in the conservative intervention groups. Type II errors for the outcomes that did not improve over time cannot be discounted. Palmer et al. [34] did not perform a sample size calculation as it was clearly identified as a pilot study with no inferential statistical analysis. It should also be noted that a direct head-to-head statistical comparison of the intervention and control groups post-treatment was not reported by Sahin et al. [36] and thus, it is not certain if that study was powerful enough to detect such a difference.

#### Follow-up

Long-term follow-up is important in demonstrating effectiveness beyond the period of active therapeutic intervention, something that is of particular relevance to life-long conditions such as syndromic hypermobility. In eight of the 11 studies, the final study outcomes were completed immediately at the end of treatment, with no long-term follow-up at all. The longest follow-up was observed in Bale et al. [27] who assessed patients at 3-month (only 6% attrition) and 12-month post-intervention (12% attrition). Completeness of follow-up is an important determinant of validity [58]. Attrition rates were generally low, with only two of the included studies exceeding 30% attrition (33% [34] and 44% [32]).

#### Outcome measures

A very wide range of outcome measures were explored, incorporating impairment, activity and participation. This is important as it evidences the comprehensive effects of the interventions investigated. Ten of the 11 studies investigated pain intensity. With the exception of Bathen et al. [28] and Palmer et al. [34], all reported statistically significant improvements in pain intensity from pre- to post-treatment.

### Strengths and limitations of the review

Key strengths of the review were the very robust search strategy, and the group processes for identifying, screening and critically appraising studies. The two reviewers experienced less agreement when using the CASP cohort study checklist than with the RCT checklist. This led to more discussion with the wider research group to agree an interpretation of those studies. Whilst this consensus might be seen as a strength, the interpretation and application of the cohort study checklist may still be open to debate. It should be acknowledged that only three of the studies included were with children, thereby limiting the application of findings to paediatric syndromic hypermobility populations. All studies included exercise as a core component of the interventions evaluated and thus the effectiveness of other conservative interventions is unknown.

### Clinical implications

This review provides valuable insight into the potential impacts of conservative management in syndromic hypermobility. Inspiratory muscle training [35], spinal stabilisation exercises [29] and a combined exercise programme (closed kinetic chain exercises and proprioception exercises) [30] all demonstrated effects that were superior to no-treatment controls. However, no clear recommendations can be made about the superiority of particular types of conservative interventions over other conservative management approaches.

Although positive findings were reported in the included studies, it is important to consider the intensity of the interventions and how well these might be integrated into healthcare delivery. For example, two of the three RCTs that demonstrated superior effects of exercise over no-treatment controls [29, 30, 35] both used rather intensive interventions. In Daman et al. [30], patients attended 3 days per week for 4 weeks, supervised by an expert physiotherapist, and in Celenay and Kaya [29], patients attended 3 days per week for 8 weeks, again supervised by an experienced physiotherapist (although that intervention was delivered as a group programme). It remains to be seen whether less therapist-intensive interventions, which employ a greater emphasis on supported self-management, can demonstrate clinical effectiveness within the context of an RCT. Interventions such as that piloted by Palmer et al. [34] might translate more easily into clinical practice as they match common physiotherapy delivery patterns, at least within a UK context [7]. Such self-management approaches are coherent with recommendations for long-term conditions [59] and enhancing capability to self-manage has been associated with lower healthcare utilisation [60].

### Recommendations for future research

Further rigorous RCTs are required. Other recommendations for future research include ensuring a priori sample size calculations; employing effective blinding and randomisation techniques to reduce the risk of bias; selecting outcome measures that capture the multidimensional impact of syndromic hypermobility; and investigating ‘whole body’ management (rather than individual joints or body areas), including conservative management approaches other than exercise.

## Conclusion

This systematic review provides weak evidence for the effectiveness of conservative management for the management of syndromic hypermobility. This is based on three small RCTs (*n* = 20–46) that demonstrated superior effects relative to no treatment on outcomes such as joint position sense, muscle endurance, pain, physical function and postural stability. However, those studies only recruited women, focused on a single area of the body and had no long-term follow-up. The review found no evidence for superior effects of specific conservative interventions when compared with other such interventions. All studies (including cohort studies) observed improvements from pre- to post-treatment in adults and children in a very wide range of impairment, activity and participation level outcomes. The reviewed evidence related to interventions that had exercise as a core component.

Evidence included in the review should be interpreted with caution due to a range of methodological limitations. There remains a need for more rigorous randomised controlled studies to better inform clinical practice. Future studies should pay particular attention to issues related to sample size, blinding, long-term follow-up, the evaluation of ‘whole body’ management (rather than individual joints or body areas) and the inclusion of adequate comparators.
